# Cyclic Fatigue Resistance and Phase Transformation Behavior of SlimShaper and SlimShaper PRO NiTi Instruments: A Mechanical and Thermal Analysis

**DOI:** 10.3390/dj14010022

**Published:** 2026-01-04

**Authors:** Cristina Scolaro, Francesco Puleio, Andrea Sili, Annamaria Visco

**Affiliations:** 1 Department of Engineering, University of Messina, 98166 Messina, Italy; cristina.scolaro@unime.it (C.S.); andreamariano.sili@unime.it (A.S.); annamaria.visco@unime.it (A.V.); 2 Department of Biomedical and Dental Sciences and Morphofunctional Imaging, University of Messina, 98122 Messina, Italy; 3 Institute for Polymers, Composites and Biomaterials—CNR IPCB, Via Paolo Gaifami 18, 95126 Catania, Italy

**Keywords:** endodontic rotary instruments, cyclic fatigue resistance, thermal treatment, phase transformation, martensite, austenite

## Abstract

**Objectives**: This study compared the cyclic fatigue resistance and the mechanical, thermal, and metallurgical characteristics of SlimShaper^®^ and SlimShaper PRO^®^ instruments. Both sequences include three instruments (ZS1–ZS3) with identical geometries, although SlimShaper PRO features an apically modified thermal treatment. **Methods**: Cyclic fatigue tests were performed using a standardized metallic guide with a 45° curvature, on six instruments of each sequence type. Fractured segments were measured, and fracture surfaces were analyzed using optical microscopy and Scanning Electron Microscopy (SEM). Elemental composition was assessed by Energy-dispersive Spectroscopy (EDS), while Differential Scanning Calorimetry (DSC) was used to determine transformation temperatures and enthalpy. **Results**: ZS1 and ZS1 PRO exhibited comparable cyclic fatigue resistance, whereas ZS2 and ZS3 showed significantly higher resistance than their PRO counterparts. SlimShaper PRO^®^ instruments fractured with segments approximately 0.5–1 mm longer. EDS confirmed that both instruments were made of NiTi, with minor differences in surface composition, while DSC demonstrated similar enthalpy values but distinct transformation ranges. At room temperature (27 °C), SlimShaper^®^ ZS2 remained predominantly martensitic, whereas ZS2 PRO could be partially austenitic, explaining its observed reduced fatigue resistance. The results of the DSC allowed to deduce the microstructure and thus the fatigue behavior at the temperature of the oral cavity. **Conclusions**: SlimShaper ZS2 and ZS3 showed significantly greater cyclic fatigue resistance than their PRO counterparts, while DSC analysis revealed distinct differences in phase transformation behavior that explain their mechanical performance.

## 1. Introduction

Cyclic fatigue of rotary instruments in curved root canals represents one of the most critical challenges in contemporary endodontics, as it directly affects both treatment safety and long-term prognosis of the tooth [1]. Starting in the 1990s, this issue led to the widespread use of nickel–titanium rotary files. Due to their superior flexibility and increased cutting efficiency, they gained popularity for their capability to shape and debride canals more efficiently than conventional stainless steel hand instruments [2].

From a materials science perspective, advances in the metallurgical processing of NiTi alloys have been central to improving instrument performance. In particular, research on heat treatments has highlighted their capacity to modify microstructure, thereby enhancing flexibility and fatigue resistance [3,4]. Clinically, these improvements translate into safer and more predictable shaping procedures, especially in anatomically challenging root canals where stress concentrations are elevated. NiTi is an intermetallic alloy composed of nickel and titanium in nearly equiatomic proportions. Its unique clinical relevance arises from the ability to change crystallographic phases in response to stress and temperature variations. Above the Austenite Start (A_S_) to Austenite Finish (A_F_) temperature range, the alloy assumes an austenitic configuration, which provides superelasticity and high cutting efficiency. Below the Martensite Start (M_S_) to Martensite Finish (M_F_) range, the alloy transforms into martensite, a softer and more ductile phase with superior cyclic fatigue resistance [5,6]. An intermediate R-phase, observed at transitional temperatures, also contributes to clinical performance by combining favorable features of both austenite and martensite [7,8]. For clinicians, this phase behavior is not a mere physical property but a determining factor for the of instrument safety and adaptability during shaping procedures. NiTi alloy behavior is primarily governed by the proportion of martensitic and austenitic phases present at clinically relevant temperatures. Heat treatments modify these phases, influencing flexibility, crack propagation patterns, and resistance to cyclic loading [9]. Modern systems exploit these treatments to achieve different mechanical behaviors across instruments within a sequence, adapting cutting efficiency and flexibility to each step of canal shaping [10]. The practical implication is that instrument selection can be tailored to the clinical scenario: austenitic instruments are more suitable for straight or mildly curved canals, while martensitic instruments are preferred for canals with severe curvatures [4].

Traditionally, manufacturers have offered endodontic instruments made from the same alloy throughout the entire sequence of instruments. However, the complex and variable nature of the canal system requires instruments with specific mechanical properties at different stages of the procedure. The first instrument in the sequence should have a thermal treatment that enhances its cutting efficiency, while subsequent instruments should be more flexible to shape a canal where the gliding path has already been established. Several manufacturers have recently introduced sequences in which each instrument is produced with different heat treatment, providing increased cutting ability for early instruments and greater flexibility for larger sizes. However, no independent investigation has clarified whether these sequential metallurgical modifications translate into predictable clinical advantages [11].

According to the manufacturer, SlimShaper PRO (Zarc4Endo, Gijón, Spain) instruments incorporate a dual thermal treatment aimed at enhancing cutting efficiency in the apical portion while maintaining flexibility in the coronal body. However, a reduction in martensitic content at the tip may also increase stiffness and susceptibility to cyclic failure, particularly in curved regions of the canal. To date, no independent study has verified whether these claims translate into meaningful differences in phase distribution or mechanical durability. Given that instrument fracture due to cyclic stress typically occurs in the curved portions of the canal—often involving the apical region—this modification could represent a mechanical disadvantage. Therefore, it is essential to investigate whether the presence of a stiffer tip, despite its improved cutting ability, compromises the instrument’s overall mechanical performance, particularly its resistance to cyclic fatigue.

This research analyzed various aspects of the mechanical, thermal, metallurgical characteristics of the SlimShaper and SlimShaper PRO instruments, and their potential impact on clinical performance. Although a recent multi-center study investigated SlimShaper PRO in terms of operator perceptions and usability, no previous studies have comprehensively characterized these instruments with respect to their combined mechanical, thermal, and metallurgical properties [12]. Therefore, the present investigation represents the first attempt to provide a detailed evaluation of mechanical and metallurgical properties of the new SlimShaper PRO instrument (Zarc4Endo, Gijón, Spain) compared to its predecessor SlimShaper^®^ (Zarc4Endo, Gijón, Spain). In clinical practice, the mechanical behavior of NiTi instruments is directly influenced by the phase composition present at intraoral temperature. The balance between martensite and austenite determines flexibility, crack propagation dynamics, and resistance to cyclic loading in regions of severe curvature. Although cyclic fatigue tests provide useful baseline information, they do not fully capture the complex thermo-mechanical interactions occurring during root canal shaping. For this reason, combining fatigue testing with advanced thermal and metallurgical analyses is clinically relevant, as it allows for a deeper understanding of how different heat treatments influence safety and performance inside the canal. The recently introduced SlimShaper PRO system has not yet undergone independent characterization, and clinicians currently lack evidence-based guidance regarding its behavior in anatomically demanding scenarios. This highlights the need for a comprehensive evaluation that links microstructural transformations to clinically meaningful mechanical outcomes.

The objective of this investigation was to conduct a comprehensive evaluation of the SlimShaper and SlimShaper PRO systems through a combination of cyclic fatigue testing, SEM-EDS surface characterization, high-resolution morphological analysis, and differential scanning calorimetry. By integrating these complementary methodologies, the study aimed to elucidate how microstructural features, phase transformation temperatures, and martensite–austenite distribution influences the mechanical behavior and expected clinical durability of the instruments in curved canal regions [13]. The null hypothesis tested was that there would be no significant differences between the two instrument systems in terms of fatigue resistance and phase transformation behavior.

## 2. Materials and Methods

### 2.1. Geometry of the Endodontic Instruments

This investigation was conducted on two commercial endodontic rotary files sets, SlimShaper (Zarc4Endo, Gijón, Spain) and SlimShaper PRO (Zarc4Endo, Gijón, Spain). Both the instruments are designed to be used in a progressive clinical sequence (ZS1 → ZS2 → ZS3). The cutting surface of SlimShaper and SlimShaper PRO (ZS1, ZS2, ZS3) instruments is made of a nickel–titanium alloy processed with three heat treatments (Gold, Pink, and Blue). According to the manufacturer, the SlimShaper sequence consists of three instruments with different apical diameters and variable tapers: ZS1 has an apical tip size 0.15 mm with a variable taper ranging from 2% to 6%; ZS2 has a tip size 0.20 mm with a variable taper ranging between 4% and 3%; and ZS3 has a tip size 0.25 mm with a variable taper of 4–3%. The SlimShaper PRO sequence maintains the same apical sizes and geometric configuration, but the manufacturer reports that the instruments undergo a modified thermal treatment applied selectively to the apical 3 mm of the file as part of the DualWire technology. However, no further technical details regarding the Gold, Pink, Blue, or DualWire thermal treatments are disclosed by the company, as these processes are proprietary and protected by industrial secrecy. These files have a total length of 31 mm.

### 2.2. Fatigue Test

To perform cyclic fatigue tests on endodontic instruments, a specially designed metal guide was used to emphasize the challenging conditions within the root canal system.

**Figure 1:**
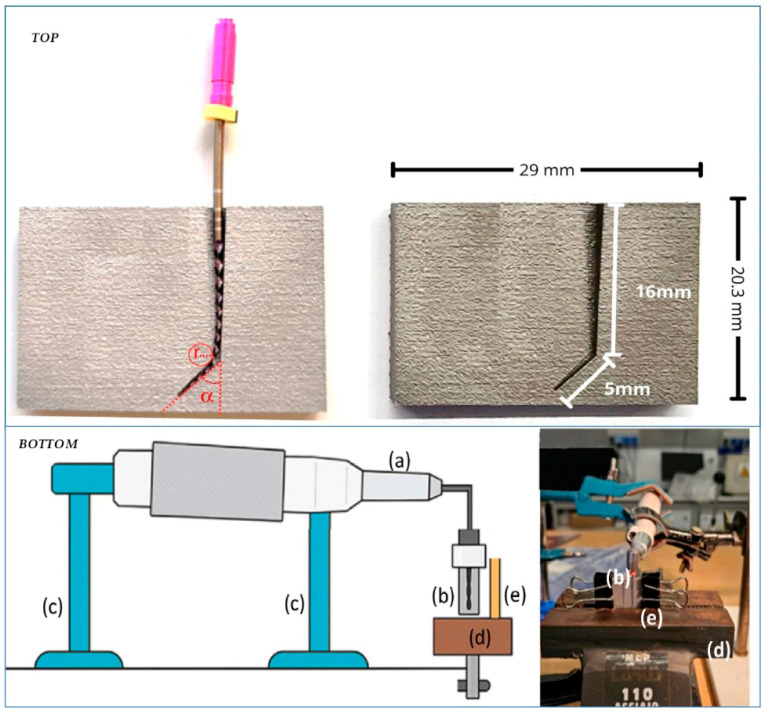
Guide with geometric details used to perform cyclic fatigue tests of the rotary files with (**left**) and without (**right**) a rotary file (**top**). Scheme (**left**) and photograph (**right**) of the experimental setup used for the cyclic fatigue test: (a) endodontic motor (rotary handpiece), (b) simulation guide, (c) two adjustable supports, (d) bench vice, and (e) Plexiglas secured to the guide with two small metal clamps (**bottom**).

This guide, manufactured using laser melting technology, measures 29 mm × 20.3 mm and reproduces the anatomical configuration of an α = 45° angled dental canal with a connection radius r~0.6 mm for a total length of 16 mm + 5 mm (Figure 1(top)). This setting was specifically designed to simulate unfavorable clinical conditions, considering that a reduced radius of curvature results in a short time to fracture under cyclic fatigue conditions [14,15] (Figure 1).

The Woodpecker E-com+ endodontic motor (Woodpecker Medical Instrument Co., Ltd., Guilin, China) was used (see detail a in Figure 1(bottom)). The rotary endodontic file was manually inserted into the rotary handpiece before each test and positioned within the simulation guide (Figure 1(bottom), detail b). To ensure stability and correct orientation, the handpiece was rigidly locked on both sides by two adjustable supports, set at different heights to align horizontally with the guide (Figure 1(bottom), detail c). The guide was held in place with a bench vice (detail d in Figure 1(bottom)), which ensured its immobility during each test cycle. To prevent the file from slipping out of the guide during rotation, a Plexiglas containment panel was attached to the front of the guide, secured to the guide with two small metal clamps (Figure 1(bottom), detail e).

Six instruments of each sequence type were included in the test: six ZS1, six ZS2, six ZS3, and six ZS1 PRO, six ZS2 PRO, and six ZS3 PRO. Instruments were examined under a 15× magnification microscope (OPMI Pro Ergo, Carl Zeiss, Oberkochen, Germany) to exclude those with manufacturing defects. The instruments were rotated at the manufacturer’s recommended settings (500 rpm; 3 Ncm). All tests were performed at ambient laboratory temperature, which was measured at 27 °C during the experimental sessions. A thin layer of lubricant was also applied inside the metallic guide to minimize friction between the file and the canal walls, in accordance with previously published protocols. At the end of each test, only the specimen was replaced, leaving the entire configuration unchanged.

#### Statistical Analysis

The mean differences and standard deviations of six samples per type were calculated with statistical software (Prism 8.0.2, GraphPad, Inc, La Jolla, CA, USA). The Shapiro–Wilk test was used for normality and lognormality tests of data, and Brown-Forsythe test for homogeneity of the variance test. A Student’s t-test was used to assess differences in time to fracture between the experimental groups. The analysis was performed to determine whether the mean fracture times differed significantly, with the level of statistical significance set at *p* < 0.05. After the cyclic fatigue test, the fractured fragments of each instrument were carefully collected and measured using a digital caliper with an accuracy of 0.01 mm. For each sequence type (ZS1, ZS2, ZS3 and their PRO counterparts), six instruments were evaluated, and the length of the fractured segment was recorded. The data were analyzed to determine whether differences existed between the SlimShaper and SlimShaper PRO instruments in terms of fragment length. The normality of the data distribution was verified using the Shapiro–Wilk test. Since the values were normally distributed, comparisons between corresponding groups (ZS1 vs. ZS1 PRO, ZS2 vs. ZS2 PRO, and ZS3 vs. ZS3 PRO) were performed using an independent-samples *t*-test with Welch’s correction for unequal variances. Statistical significance was set at *p* < 0.05.

### 2.3. SEM-EDS Measurements and Optical Microscopy Observations

The chemical–morphological analysis by SEM-EDS (Scanning Electron Microscopy with Energy-Dispersive X-Ray Spectroscopy) and optical microscopy (OM) represent a crucial phase of our study, as it allows us to investigate in detail the surface morphology and chemical composition of the SlimShaper^®^ and SlimShaper PRO^®^ endodontic instruments [16]. To determine the composition of the NiTi alloys of the endodontic instruments, a microscope (Carl Zeiss Crossbeam 540 Microscopy, GmbH, 07745 Jena, Germany) with an accelerating voltage of 5 kV was used. Image magnifications were 300× and 2000×. The system is equipped with an Oxford Instruments XMaxN 150 EDS detector and a Nordlys Max EBSD system. This allowed for the acquisition of three-dimensional datasets containing chemical and crystallographic data. To observe before and after fracture of the endodontic files, optical microscopy (OM) images were acquired using a Hirox digital microscope mod. KH8700 (Hirox, Tokyo, Japan) mounting a 103 MX(G)-5040Z lens.

### 2.4. Differential Scanning Calorimetry

To investigate the phase transformations in detail, two specimens were cut from each endodontic file subjected to fatigue tests: the fractured tip, and the middle conical threated part. Phase transformations of each specimen were studied by Differential Scanning Calorimetry (DSC) using a Mettler Toledo-DSC1 instrument with liquid nitrogen cooling. The heating and cooling regime of each specimen was as follows: first, heating up to 150 °C, followed by cooling down to −20 °C to achieve the corresponding cooling curve. After keeping at −20 °C for 5 min, the sample was heated up to 150 °C to achieve the corresponding heating curve. The cooling and heating rates during thermal cycling were equal to 10 °C/min. The transformation temperatures were determined by the intercept method of the extrapolated tangent.

## 3. Results

### 3.1. Fatigue Test

No instruments were excluded from the analysis, as none showed manufacturing defects under 15× magnification. Average times to failure due to cyclic fatigue rotation rates and number of cycles to failure for the SlimShaper^®^ (ZS1, ZS2, ZS3) and SlimShaper PRO^®^ instruments (ZS1 PRO, ZS2 PRO, ZS3 PRO) are summarized in Table 1.

Based on the obtained data, the percentage Δ between the number of cycles to failure (N) of the SlimShaper^®^ and SlimShaper^®^ PRO instruments was calculated according to the following formula:

D [%]=100×N (SlimShaper) − N (SlimShaperPRO)N (SlimShaper) 

The comparison between ZS1 and ZS1Pro was statistically insignificant. A significant difference was found in the comparison between ZS2 and ZS2 Pro and ZS3 and ZS3 Pro. The analysis of fractured fragment lengths revealed distinct differences between the SlimShaper^®^ and SlimShaper PRO^®^ instruments. The mean fractured fragment length for SlimShaper^®^ ZS1 was 4.32 ± 0.69 mm, while for SlimShaper PRO^®^ ZS1 it was 4.82 ± 0.41 mm; the difference was not statistically significant (*p* = 0.1599). SlimShaper^®^ ZS2 instruments showed a mean fragment length of 4.34 ± 0.12 mm, compared to 5.19 ± 0.20 mm for SlimShaper PRO^®^ ZS2, with a statistically significant difference (*p* = 0.0001). Similarly, SlimShaper^®^ ZS3 instruments fractured with an average segment length of 4.22 ± 0.07 mm, while SlimShaper PRO^®^ ZS3 exhibited longer fractured segments averaging 5.01 ± 0.37 mm (*p* = 0.0028).

### 3.2. Morphological Investigation

**Figure 2:**
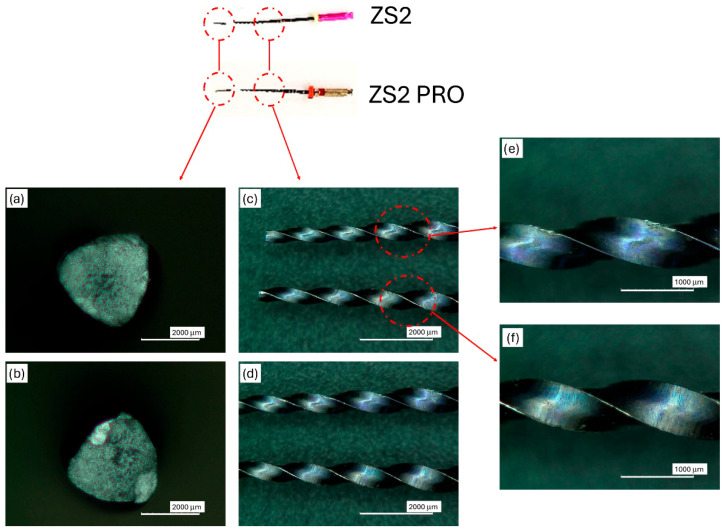
Optical micrographs taken after the fatigue test: fracture surface of ZS2 (**a**) and ZS2 PRO instrument (**b**); ZS2 and ZS2 PRO instrument in the fracture zone (**c**); ZS2 and ZS2 PRO far the fracture area (**d**); details of the threaded stems near the fracture zone of ZS2 (**e**) and ZS2 PRO instrument (**f**).

**Figure A1:**
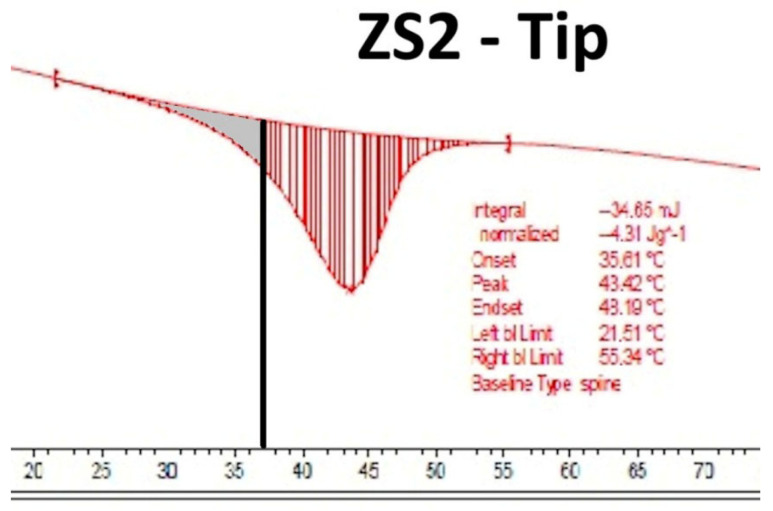
Example of integration from As up to 37 °C of the enthalpic peak to determine the fraction of Austenite for ZS2 instrument, in its tip.

Progressive magnifications were acquired in the central and peripheral regions of the fracture to obtain a comprehensive view of the crack propagation mode. Figure A1 shows the ZS2 and ZS2 PRO instruments observed under the optical microscope before carrying out the cyclic fatigue test. Figure 2 shows in detail the investigations carried out under the optical microscope after the fatigue test: the fracture surface of the ZS2 and ZS2 PRO instruments, the images of these instruments near and far from the fracture zone, and finally the details of the threaded stems near the fracture zone.

The fracture surface of ZS2 (Figure 2a) appears relatively homogeneous, with an overall fine and evenly distributed texture. A slightly more granular and irregular central zone is evident, which can be interpreted as a crack propagation area. The thin parallel and curvilinear profiles visible near one edge suggest a possible fracture initiation point due to contact with the guide walls. The fracture area has smooth edges and shows no signs of instantaneous overload fracture, confirming progressive failure, consistent with the high mean number of cycles to failure recorded. The fracture surface of ZS2 PRO (Figure 2b) appears sharper and more irregular, with more contrasting surfaces and the presence of flat areas with abrupt transitions. Some areas have a more compact and reflective appearance, suggesting reduced energy dissipation during failure.

### 3.3. SEM-EDS Analysis

**Figure 3:**
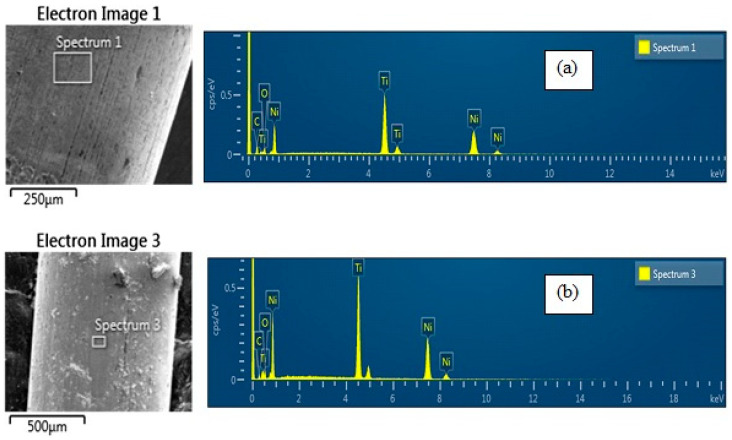
SEM micrograph and EDS peaks of the SlimShaper (**a**) and SlimShaper PRO (**b**).

Detailed observations at various points on the SlimShaper and SlimShaper PRO instruments were performed, focusing specifically on the threaded shaft and the groove connection (Figure 3).

These areas were chosen to assess any differences in material composition and identify possible variations in elemental distribution through EDS analysis.

The elemental composition analysis revealed distinct differences between the two instruments. SlimShaper exhibited a composition of 14.38% carbon, 9.10% oxygen, 41.64% nickel, and 34.87% titanium. In contrast, SlimShaper PRO showed a reduced carbon (5.24%) and oxygen (7.24%) content, accompanied by higher nickel (47.03%) and titanium (40.14%) levels. As can be seen from the collected data, the first sample analyzed consists primarily of a NiTi alloy, confirming the nature of the material used to manufacture the instruments. In addition to the two main elements, the analysis revealed the presence of detectable quantities of carbon and oxygen. The presence of oxygen can be attributed to surface oxidation phenomena, resulting from manufacturing processes or any surface treatments, on the other hand, could derive either from contamination during the production process or from organic residues deposited on the surface of the instrument. EDS spectroscopic analysis revealed that the SlimShaper PRO sample is still composed of a NiTi alloy, but with a significant difference compared to the previous sample: the percentages of carbon and oxygen are lower. This variation could indicate a different surface composition, or the presence of a specific treatment aimed at reducing oxidation or other chemical phenomena on the surface of the instrument.

### 3.4. DSC Analysis

**Figure 4:**
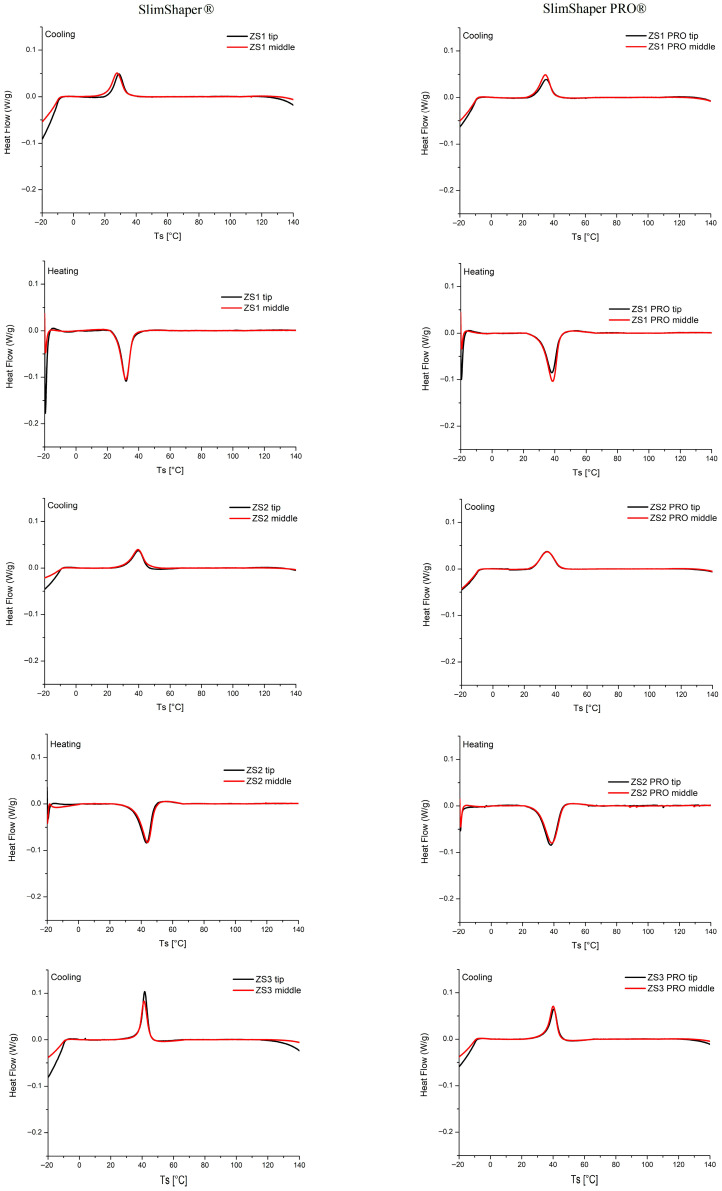

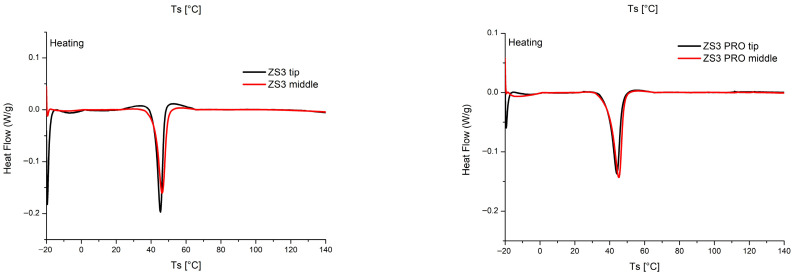
Cooling and heating curves for the tip (black) and middle (red) part of the ZS1, ZS2, ZS3 SlimShaper^®^ and SlimShaper PRO^®^ instruments.

The ZS1, ZS2 and ZS3 and the PRO-sequence instruments were subdivided into two parts (tip and body of the instrument) to determine the transformation temperatures by DSC analysis. The cooling stage and heating stage of each part are reported in Figure 4 and Figure A1.

These curves are characterized by the presence of an exothermic peak during cooling and an endothermic peak during heating, demonstrating the occurrence of the transformations from austenite to martensite and its reverse, respectively.

The transformation temperatures were evaluated by the intercepts of the extrapolated tangents at the onset and endset of each peak. On cooling, the onset and endset temperatures represent, respectively, M_S_ and M_F_; while, on heating, they represent, respectively, A_S_ and A_F_. These values together with the enthalpy peak areas are summarized in Table 2.

The following considerations can be deduced from Table 2:

- The normalized peak area, representing the transformation enthalpy, is essentially the same for each part of the endodontic instruments, being in the range between 4 and 5 mJ/g.
- The tip and the middle part of each ZS1, ZS2, ZS3 SlimShaper^®^ and SlimShaper PRO^®^ instrument do not show appreciable differences in the transformation temperatures; therefore, it can be deduced that each instrument, presenting homogeneous characteristics, has not undergone differentiated heat treatments.
- Room temperature (27 °C) is slightly higher than A_S_ (equal to about 26 °C) only for the ZS1 SlimShaper^®^ instrument, which could be partially austenitic. In all other cases A_S_ ≥ 30 °C, excluding the formation of austenite at room temperature.
- Room temperature (27 °C) is higher than M_F_ (equal to about 23 °C) for the ZS1 SlimShaper^®^ instrument and slightly higher than M_F_ (equal to about 26 °C) for the ZS2 SlimShaperPRO^®^ instrument, which could be partially austenitic. In all other cases M_F_ ≥ 27 °C, excluding the formation of austenite at room temperature. This finding indicates that both instruments exhibit homogeneous characteristics along their length and have not undergone differentiated heat treatments. Based on the transformation temperature values reported in Table 2, the microstructures at room temperature can be deduced as summarized in Table 3.

The values at body temperature were consequently obtained, the temperature at which these instruments rotate during clinical procedures (Table 4). The percentage of austenite at temperature (37 °C) can be calculated from the heating curves as the following ratio (see, for example, the enthalpy peak shown in Figure A1):

$$Austenite\;\left(\%\right)=100\times \frac{Peak\;area\;integrated\;from\;AS\;to\;37\;{}^{\circ}C}{Total\;peak\;area}$$

## 4. Discussion

In comparative studies, differences in mechanical properties are often influenced by variations in design parameters such as cross-sectional shape, taper, or flute geometry [17,18]. In the present investigation, however, both SlimShaper^®^ and SlimShaper PRO^®^ instruments are geometrically identical, sharing the same cross-section, taper, and flute design. Consequently, the metallurgical phase is the only distinguishing factor between the two systems. This unique condition allows us to attribute any observed differences in cyclic fatigue resistance, exclusively to the effect of the microstructure, without the confounding influence of geometric variables.

### 4.1. Fatigue Test

The cyclic fatigue test was performed because, according to the manufacturer, the SlimShaper PRO^®^ instruments are designed with an apical portion that contains a lower martensitic content, resulting in a stiffer and more austenitic tip. At room temperature, the cyclic fatigue behavior of SlimShaper^®^ ZS1 and SlimShaper PRO^®^ ZS1 appeared comparable, suggesting that for smaller apical sizes the applied thermal modifications do not markedly affect fatigue performance. In contrast, for ZS2 and ZS3 instruments, the conventional SlimShaper^®^ files exhibited a more favorable fatigue behavior compared with their PRO counterparts. This trend may reflect differences in phase transformation characteristics and microstructural features, which could become more influential as instrument size and taper increase, ultimately affecting stress accumulation and crack propagation during cyclic loading. This outcome can be explained by the fact that the microstructures resulting from the heat treatment applied to the first instruments of the sequence (ZS1) do not differ to the point of generating clinically relevant changes in fatigue resistance, while the treatments applied to the ZS2 and ZS3 instruments lead to a reduction in the cyclic fatigue resistance of the SlimShaperPRO^®^ instruments. This result experimentally confirms the disadvantage in the fatigue behavior of the austenitic microstructure highlighted in the literature [8,19].

Another important aspect concerns the length of the fractured segment. On average, the fractured portion of the SlimShaper PRO^®^ instruments was 0.5 to 1 mm longer than that of the SlimShaper^®^ instruments. From a clinical perspective, a longer fractured fragment may represent a considerable advantage: in the event of instrument separation inside the root canal, the retrieval of a longer fragment is technically easier and carries a higher probability of success compared to the removal of a shorter fragment [20].

### 4.2. Morphological Investigation

To integrate and complete the observations obtained, a morphological investigation of the fracture surfaces of the ZS2 and ZS2 PRO instruments was conducted. The analysis focused on the fracture zones of the two instruments, with the aim of investigating the failure mechanisms, and identifying any recurring morphological characteristics that could indicate the predominant type of stress (wear, torsion, sudden overload).

Microscopy confirmed that the fractured instruments exhibited the typical signs of cyclic fatigue failure, including crack initiation at the periphery of the cross-section, slow crack propagation with smooth regions, and striations indicating the progressive advancement of the crack under repeated tensile stresses. The presence of peripheral crack initiation sites and smooth propagation areas strongly supports that the observed fractures were the result of cyclic loading rather than torsional stress [21,22]. The crack propagation patterns observed in this study are consistent with the metallurgical mechanisms described in previous reviews on thermal-treated NiTi instruments [23].

Overall, the optical microscopy images highlighted clear differences between the two instruments analyzed: ZS2 exhibited a more uniform and finely textured fracture surface, compatible with progressive crack propagation, whereas ZS2 PRO revealed sharper transitions and areas of brittle-like morphology. The sharper and more irregular fracture surfaces observed in ZS2 PRO may be indicative of a more brittle fracture mechanism and accelerated crack propagation, a behavior that has been previously associated with predominantly austenitic NiTi microstructures. This interpretation is consistent with the reduced cyclic fatigue resistance observed for PRO instruments and with the phase transformation trends identified by DSC analysis. These findings suggest that the PRO tip, compared to the conventional version, has a reduced capacity for plastic deformation, favoring faster crack advance once initiated. This observation correlates with the significantly shorter fracture time observed in the test, suggesting that the PRO instrument is mechanically stiffer and less deformable at the apical portion, while the main body likely shares a similar mechanical nature with the non-PRO counterpart. The different morphology of the ZS2 and ZS2 PRO surfaces are consistent with what would be expected based on the fatigue test results. While the ZS2 instrument shows signs of stable propagation under cyclic stress (Figure 2f), the fracture surface of ZS2 PRO suggests a more rapid failure (Figure 2e), in agreement with the reduced number of cycles to failure.

### 4.3. SEM-EDS Analysis

In order to further characterize these instruments, an SEM-EDS analysis was performed. SEM-EDS analysis allowed us to examine in detail the distribution of chemical elements present in the samples, providing essential data to understand any differences between the two, groups of instruments. However, its relevance is still dubious [24,25].

Alapati et al. highlighted that variations in the Ni and Ti ratios of wire blanks, along with differences in machining processes, may alter the metallurgical properties of NiTi instruments and consequently affect their mechanical behavior [26]. In this regard, recent studies demonstrated that in Ni rich alloys aging treatments at 400 °C leads to Ni_4_Ti_3_ precipitation within the NiTi matrix, favoring the R-phase formation [8]. The presence of the R-phase ensures good SME and SE effect, as well as cyclic stability [7]. Furthermore, by adding less than 1% of alloying elements to Nitinol, the so-called M-wire, containing austenite with small amounts of M-phase and R-phase, and characterized by improved fatigue resistance, can be obtained [10]. Nevertheless, the specific influence of nickel weight percentage remains unclear, as no studies have investigated this variable while keeping all other factors—such as instrument design and heat treatment—unchanged. This makes it difficult to establish whether mechanical performance is determined primarily by the Ni/Ti ratio or, more plausibly, by the combined effect of multiple interrelated factors.

### 4.4. DSC Analysis

From a clinical perspective, the interpretation of DSC data must always be contextualized to the actual conditions of use of endodontic instruments, namely intraoral temperature and the mechanical stress generated by continuous contact with dentinal walls [27,28]. The temperature-dependent transformation behavior detected by DSC aligns with recent evidence demonstrating that heat-treated NiTi alloys show significant variations in mechanical performance at 37 °C [29,30].

In this study, the normalized peak area, representing the transformation enthalpy, was essentially the same for all parts of the SlimShaper^®^ and SlimShaper PRO^®^ instruments, with values consistently between 4 and 5 mJ/g. In particular, it can be observed that, for each ZS1, ZS2, ZS3 SlimShaper^®^ and SlimShaper PRO^®^ instrument, the DSC curves of the tip and the middle part are superimposable (Figure 4). This result was expected for the SlimShaper^®^ sequence, as the manufacturer declares that all instruments are produced from a single alloy. Conversely, it was not anticipated for the SlimShaper PRO^®^ instruments, which are reported to undergo two distinct thermal treatments along their length.

The transformation temperature ranges, however, revealed important distinctions between the two instruments. The results of DSC analysis show that the ZS1 instrument is fully austenitic at 37 °C, while the ZS1 PRO instrument maintains a significant percentage of martensite. Therefore, the ZS1 PRO instrument should have better cyclic fatigue behavior than the non-PRO one.

For ZS2, the M_S_–M_F_ and A_S_–A_F_ intervals were approximately 45–31 °C and 36–49 °C, respectively. Thus, at room temperature the alloy is fully martensitic, and at oral temperature (37 °C) only a small portion of martensite begins to transform into austenite. In contrast, ZS2 PRO showed M_S_–M_F_ and A_S_–A_F_ ranges of about 42–26 °C and 30–46 °C, respectively, indicating that at 37 °C a larger fraction of the alloy has already transformed into austenite. The percentage of austenite in the ZS2 instrument is between 12 and 18%, therefore it can be considered predominantly martensitic at 37 °C. Conversely, in the ZS2 PRO instrument the percentage of austenitic is between 38 and 45% and, for this reason, worse fatigue behavior is to be expected at the intraoral temperature.

Both the ZS3 and ZS3 PRO instrument are fully martensitic at the intraoral temperature; therefore, it is not possible to make predictions on which of the two instruments may have better performance under cyclic fatigue.

Clinically, these findings suggest that, although transformation enthalpy values are similar, the phase composition at intraoral temperature differs considerably between the two instruments. ZS2 remains predominantly martensitic at 37 °C, ensuring higher flexibility and superior cyclic fatigue resistance; importantly, these data are confirmed by the results of the cyclic fatigue test, which demonstrated that ZS2 instruments exhibited significantly greater resistance than ZS2 PRO. ZS2 PRO, on the other hand, is already partially transformed into austenite at the same temperature, making it stiffer and more reactive, with potentially improved cutting efficiency but reduced durability under cyclic loading.

The results obtained from the comparison of each pair of instruments indicate that the PRO version is indeed more austenitic than the conventional one; however, this difference is not confined to the tip but involves the entire instrument.

While enthalpy provides useful complementary information for understanding the energetic dynamics of the martensite↔austenite transition, it cannot be considered a direct predictor of clinical performance. The decisive factor remains the interaction between enthalpy, the proportion of martensite and austenite present at intraoral temperature, and the mechanical stresses applied during root canal shaping. In this context, DSC analysis confirms that the observed differences in fatigue resistance between SlimShaper^®^ and SlimShaper PRO^®^ are mainly attributable to their distinct phase transformation behavior rather than differences in transformation enthalpy. Considering that the instruments examined in the present article would both be austenitic at 60 °C, it is worth evaluating the percentages of the phases at the intraoral temperature equal to 37 °C, a crucial value for the onset of the austenitic transformation.

### 4.5. Clinical Implications

From a clinical perspective, the experiments performed in this study demonstrated that SlimShaper^®^ ZS1 files exhibited comparable cyclic fatigue resistance to their PRO counterparts, whereas ZS2 and ZS3 showed significantly greater resistance than the corresponding PRO instruments. Based on these findings, clinicians who prioritize cyclic fatigue resistance and the ability to safely use instruments across multiple canals are advised to begin shaping with the SlimShaper PRO^®^ ZS1—benefiting from its sharper apical tip without compromising fatigue resistance—and to continue the sequence with SlimShaper^®^ ZS2 and ZS3. Conversely, in cases where enhanced cutting efficiency is required, such as canals with narrow or partially calcified anatomy, increased dentin sclerosis, reduced initial patency, or when more effective coronal and middle-third enlargement is needed during the early shaping phases the use of the complete SlimShaper PRO^®^ sequence may be recommended, although clinicians should be aware of its reduced cyclic fatigue resistance [6,9,31].

Moreover, although SlimShaper PRO^®^ instruments are designed with different thermal treatments along their length, the present findings suggest that the microstructer is homogeneously distributed throughout the entire instrument rather than being differentiated between the tip and the body.

### 4.6. Limitations

This study presents some limitations that should be acknowledged. All tests were performed under controlled in vitro conditions using a metallic canal with a standardized curvature. Although this configuration ensures reproducibility, it cannot fully reproduce the complex clinical environment, where factors such as temperature variation, canal humidity, and interaction with irrigating solutions may influence the mechanical response of nickel–titanium instruments. The artificial canal employed for cyclic fatigue testing reproduced a single curvature and radius, which does not account for the multiple curvatures and irregular cross-sections often present in natural root canals. Furthermore, the instruments were tested under static conditions, without the dynamic axial movements that occur during clinical shaping, which could modify stress distribution and fatigue behavior. Moreover, the standardized canal does not replicate the anatomical constraints imposed by the access cavity, which may further influence instrument mechanics and clinical behavior. Another limitation is that torsional fatigue was not assessed. In clinical conditions, endodontic instruments are simultaneously exposed to cyclic and torsional stresses; therefore, evaluating torsional resistance could provide additional insight into mechanical behavior. Nonetheless, the present investigation combined cyclic fatigue testing with SEM-EDS analysis, high-resolution morphological evaluation, and DSC assessment, allowing a comprehensive characterization of the alloy and its phase-dependent mechanical response. Although only six instruments per group were tested, no variability or manufacturing irregularities were detected among samples during the preliminary microscopic inspection. As these instruments are industrially produced with standardized and highly controlled manufacturing processes, the likelihood of significant inter-instrument variability is low; nonetheless, larger sample sizes could further confirm these observations in future studies. Although only six instruments per group were tested, this sample size is in line with previous cyclic fatigue studies on NiTi files and, given the large differences observed between groups, was adequate to detect clinically relevant variations in fatigue behavior. Nevertheless, we acknowledge that such a sample is not sufficient to identify extremely rare manufacturing defects, which would require much larger datasets.”

### 4.7. Strengths of the Study

A major strength of this investigation is that it goes far beyond the traditional cyclic fatigue assessment commonly used in NiTi research. In addition to fatigue testing, we performed multiple advanced metallurgical analyses—including differential scanning calorimetry, SEM-EDS evaluation, and high-resolution morphological assessment—to characterize the instruments in a comprehensive and multidimensional manner. This integrated approach provides a deeper understanding of how phase transformation behavior and alloy microstructure influence the mechanical performance of SlimShaper and SlimShaper PRO instruments under clinically relevant conditions. To date, no published studies have directly compared SlimShaper and SlimShaper PRO instruments. This lack of comparative data highlights the novelty and relevance of the present investigation, which represents the first independent evaluation of the mechanical and metallurgical differences between the two systems.

### 4.8. Further Research

Future investigations should aim to evaluate the performance of these instruments under experimental conditions that more closely reproduce the complexity of the clinical setting. In particular, dynamic cyclic fatigue testing incorporating axial motion, testing at body temperature, and the use of anatomically realistic canals with variable curvature and diameter could provide additional insights into instrument behavior under clinically relevant stresses. Moreover, the combined evaluation of cyclic fatigue and torsional loading, as well as testing in the presence of commonly used irrigants and lubricants, may better reflect the multifactorial mechanical challenges encountered during root canal shaping. Finally, ex vivo studies on extracted teeth and well-designed clinical studies would be useful to validate the in vitro findings and to further assess the safety, durability, and shaping efficiency of these instruments in real clinical conditions.

## 5. Conclusions

Within the limitations of this in vitro study, cyclic fatigue confirmed that SlimShaper^®^ ZS2 and ZS3 exhibit significantly greater resistance than their PRO counterparts, while ZS1 and ZS1 PRO showed similar performance. DSC analysis revealed the possible presence of austenite in the ZS2 SlimShaperPRO^®^ instrument, explaining its worse fatigue behavior at room temperature. Furthermore, at the intraoral temperature, martensite is present in higher percentage in the non-PRO ZS2 instrument, ensuring greater flexibility and durability; whereas the SlimShaper ZS2 PRO^®^ instruments, being more austenitic, is stiffer and potentially more efficient in cutting but more prone to fatigue failure. From a clinical perspective, SlimShaper^®^ instruments could be preferable in anatomically complex or severely curved canals where fatigue resistance is critical, while SlimShaper PRO^®^ instruments could be advantageous in cases requiring greater cutting efficiency, in particular for the ZS2 instrument provided that clinicians remain mindful of their reduced cyclic fatigue resistance.

## Figures and Tables

**Table 1:** Heat treatment, mean times to failure due to cyclic fatigue, calculated number of cycles to failure, relative difference (D) for SlimShaper^®^ and SlimShaper PRO^®^ and *p*-values.

SlimShaper					SlimShaper PRO			
	Mean Fracture Time [s]	Mean Number of Cycles Before Fracture	Treatment	Mean Fracture Time [s]	Mean Number of Cycles Before Fracture	Treatment	D [%]	*p* Value
**ZS1**	102.8 ± 22.39	856.6	Gold	105.83 ± 16.76	881.91	Gold/Silver	−5.99	0.7981
**ZS2**	59.5 ± 16.31	495.8	Pink	31.00 ± 5.18	258.3	Pink/Gold	72.86	0.0022
**ZS3**	67.0 ± 14.17	558.3	Blue	39.67 ± 5.47	330.58	Blue/Gold	64.94	0.0013

**Table 2:** Transformation temperatures and enthalpies, on cooling and heating, of the tip and middle part of each ZS1, ZS2, ZS3 SlimShaper^®^ and SlimShaper PRO^®^ instruments. Ms = Martensite Start; M_F_ = Martensite Finish; Mp = Martensitic peak; Na = Normalized peak area; As = Austenite Start; A_F_ = Austenite Finish.

	Cooling (°)					Heating (°)				
	M_S_ [°C]	M_F_ [°C]	Mp [°C]	Peak Area [mJ]	Na [mJ/g]	A_S_ [°C]	A_F_ [°C]	Ap [°C]	Peak Area [mJ]	Na [mJ/g]
	**ZS1**									
**Tip**	33.49	23.79	28.95	13.21	3.22	26.23	36.14	31.93	17.64	4.30
**Middle**	33.18	22.33	27.70	27.8	3.49	25.62	36.27	31.58	36.65	4.60
	**ZS1 PRO**									
**Tip**	40.47	28.26	35.28	20.07	3.39	31.13	43.17	38.10	24.45	4.13
**Middle**	39.97	27.75	34.62	39.02	4.42	31.55	43.12	38.74	41.88	4.74
	**ZS2**									
**Tip**	45.65	31.89	39.70	35.16	4.37	35.61	48.19	43.42	−34.65	−4.31
**Middle**	45.83	31.59	39.44	37.65	5.07	35.99	49.00	43.95	−36.19	−4.89
	**ZS2 PRO**									
**Tip**	42.11	26.39	34.35	19.54	3.94	29.94	45.17	38.12	−23.50	−4.74
**Middle**	42.45	26.13	34.70	32.64	4.06	30.06	45.67	38.76	−38.49	−4.79
	**ZS3**									
**Tip**	44.89	38.68	41.62	21.88	5.35	41.64	47.79	45.42	24.02	5.87
**Middle**	45.04	37.83	41.23	62.59	4.87	41.82	49.51	46.36	70.37	5.48
	**ZS3 PRO**									
**Tip**	44.40	35.76	40.54	34.17	4.71	38.82	47.27	44.07	36.75	5.06
**Middle**	44.03	35.38	39.98	68.60	5.05	39.17	48.30	45.19	78.81	5.80

**Table 3:** Microstructures at 27 °C, according to the transformation temperatures shown in Table 2.

Instrument Code	Investigated Part	Microstructures at 27 °C
ZS1	Tip	Martensitic/partially austenitic
	Middle	Martensitic/partially austenitic
ZS1 PRO	Tip	Martensitic
	Middle	Martensitic
ZS2	Tip	Martensitic
	Middle	Martensitic
ZS2 PRO	Tip	Martensitic/partially austenitic
	Middle	Martensitic/partially austenitic
ZS3	Tip	Martensitic
	Middle	Martensitic
ZS3 PRO	Tip	Martensitic
	Middle	Martensitic

**Table 4:** Total peak area, area integrated from *A_S_* to 37 °C, and Austenite and Martensite percentages at 37 °C for the tip and middle part of the ZS1, ZS2, ZS3 SlimShaper^®^ and SlimShaper PRO^®^ instruments.

Instrument Code	Investigated Part	Total Peak Area [mJ/g]	Area Integrated from A_S_ to 37 °C [mJ/g]	Austenite at 37 °C [%]	Martensite at 37 °C [%]
ZS1	Tip	4.30	4.30	100	0
	Middle	4.60	4.60	100	0
ZS1 PRO	Tip	4.13	1.34	32.42	67.58
	Middle	4.74	2.44	51.45	48.55
ZS2	Tip	4.31	0.80	18.52	81.48
	Middle	4.89	0.60	12.18	87.82
ZS2 PRO	Tip	4.74	2.12	44.79	55.21
	Middle	4.79	1.98	41.30	58.70
ZS3	Tip	5.87	0	0	100
	Middle	5.48	0	0	100
ZS3 PRO	Tip	5.06	0	0	100
	Middle	5.80	0	0	100

## Data Availability

The original contributions presented in this study are included in the article. Further inquiries can be directed to the corresponding author.

## Abbreviations

The following abbreviations are used in this manuscript:

A_S_	Austenite Start
A_F_	Austenite Finish
M_S_	Martensite Start
M_F_	Martensite Finish
SEM-EDS	Scanning Electron Microscopy with Energy-Dispersive X-Ray Spectroscopy
OM	optical microscopy
NiTi	Nickel–Titanium
Mp	Martensitic peak
DSC	Differential Scanning Calorimetry
